# Understanding the Density and Distribution of Restaurants in Los Angeles County to Inform Local Public Health Practice

**DOI:** 10.5888/pcd16.180278

**Published:** 2019-01-17

**Authors:** Lauren N. Gase, Gabrielle Green, Christine Montes, Tony Kuo

**Affiliations:** 1 Division of Chronic Disease and Injury Prevention, Los Angeles County Department of Public Health, Los Angeles, California; 2 Spark Policy Institute, Denver, Colorado

## Abstract

**Introduction:**

To describe the potential reach of restaurant-based strategies that seek to improve the healthfulness of menu options, it is important to understand the local restaurant environment, including the extent to which restaurants subject to policy mandates are located in communities disproportionately affected by diet-related diseases.

**Methods:**

This cross-sectional study examined the restaurant environment in Los Angeles County, a large jurisdiction with diverse geographic and socioeconomic characteristics, specifically 1) the number and characteristics of restaurants; 2) the association between neighborhood sociodemographics and restaurant density; and 3) the association between neighborhood sociodemographics and restaurant characteristics, including chain status (large chain, small chain, independent restaurant). Data sources were 1) industry data on restaurant location and characteristics (N = 24,292 restaurants) and 2) US Census data on neighborhood sociodemographics (N = 247 neighborhoods). We conducted descriptive and bivariate analyses at the restaurant and neighborhood level.

**Results:**

Countywide, only 26.5% of all restaurants were part of a large chain (a chain with ≥20 locations). We found positive associations between restaurant density and neighborhood proportions of non-Hispanic white residents and residents with more than a high school education. We found limited support to suggest a greater density of large chains in neighborhoods with lower socioeconomic status.

**Conclusion:**

Results highlight the potentially limited reach of strategies targeting chain restaurants and point to the importance of including small chain restaurants and independent restaurants in public health efforts to improve the healthfulness of restaurants. Understanding where restaurants are in relation to priority populations is a critical step to planning strategies that address diet-related disparities.

**Summary**

**What is already known on this topic?**

Studies have found associations between neighborhood sociodemographics and restaurant density and restaurant type, often categorizing restaurants as “fast food” or “full service.”

**What is added by this report?**

This study provides insight into the potential reach of program or policy strategies that target chain restaurants. To inform local public health planning, we examined where restaurants, including chain restaurants, were located in Los Angeles County, California.

**What are the implications for public health practice?**

Results highlight the limited reach of strategies targeting chain restaurants. Other jurisdictions can build on the methods used in our study to enhance understanding of their own local landscape.

## Introduction

As consumers purchase more meals away from home than previously, strategies to increase the healthfulness of food and beverages offered at restaurants have garnered increased attention (1,2). Examples of restaurant-focused policies include menu labeling (3), ordinances banning restaurants from giving away free toys with children’s meals unless the meal meets nutritional guidelines (4), and ordinances mandating that restaurants serve healthy beverages as the default option (5). Examples of voluntary initiatives include increasing the number of healthy options, offering smaller portion sizes, or offering attractive pricing for healthy options (6,7).

Both mandatory policies and voluntary initiatives have typically targeted chain restaurants: restaurants that do business under the same name and offer substantially the same menu items. National menu labeling policy included in the Patient Protection and Affordable Care Act applies only to restaurants with 20 or more locations (3). Voluntary initiatives also frequently aim to engage, and often have high rates of participation from, chain restaurants (8,9). Chains have generally been the focus of restaurant-based strategies for 3 reasons. First, chains are believed to pose a higher risk to consumers than other types of restaurants: chains usually offer foods that have minimal nutritional value, are widely accessible, and/or have many repeat customers (10). Second, the cost of implementing initiatives is lower for chains than for other types of restaurants, because chains can more easily absorb the costs associated with healthy eating strategies, such as the costs of nutritional analyses (11,12). Third, chains are better equipped than other types of restaurants to adhere to the requirements of initiatives, such as following standardized recipes (13).

To advance restaurant-based strategies at the local level, it is important to understand where restaurants are located, including the extent to which restaurants subject to policy mandates are in communities disproportionately affected by diet-related diseases. Although previous studies examined neighborhood sociodemographics associated with restaurant density, most categorized restaurants as “fast food” or “full service” as opposed to “chain” or “non-chain” (14–16). Although useful, such studies provide little insight into the potential reach of program or policy strategies that target chain restaurants. To inform local public health planning, we aimed to describe where restaurants, especially chain restaurants, are located in Los Angeles County, California.

## Methods

This cross-sectional study, conducted in late 2016 and early 2017, sought to answer the following questions: 1) What is the current number and what are the characteristics of restaurants in Los Angeles County?; 2) What neighborhood sociodemographic characteristics are associated with a greater presence of restaurants (in general)?; and 3) What neighborhood sociodemographic characteristics are associated with restaurant characteristics, in particular the density of chain restaurants? Although our primary goal was to inform local decision making, we hope that other jurisdictions can build on the methods used in our study to enhance understanding of their own local landscape.

We used data from 2 sources: industry data on restaurant characteristics and US Census data on neighborhood characteristics. Information on restaurant characteristics was provided by a market research firm that tracks restaurant industry trends nationally. The firm defines a restaurant as a location whose primary purpose is to serve food away from home on an open, commercial basis. Twenty market research staff members at this market research firm work daily on data validation through a multitiered strategy that includes monthly searches for social media reviews, automated telephone calls to check a restaurant’s telephone connectivity, and direct outreach though email and telephone surveys.

The primary restaurant characteristic of interest was chain status. We determined chain status on the basis of the number of restaurant locations that conducted business using the same name, that offered similar menu items, and whose link could be verified via internet or telephone. To understand the potential reach of strategies at the national and local level, we created 2 variables for chain status, one based on the number of locations nationally and one based on the number of locations in Los Angeles County. We classified restaurants as independent (single location), small chain (2–19 locations), or large chain (≥20 locations), in accordance with previous research and policy scope (eg, the menu labeling policy included in the Patient Protection and Affordable Care Act) (17,18).

We also categorized restaurants according to industry market segment and cuisine type. Industry market segment was coded by the market research firm as 1) quick service (patrons order at counter; meals typically under $10), 2) fast casual (patrons order at counter; slightly higher price point than quick service), 3) midscale dining (offers sit-down/full table service; typically does not serve alcohol; entrée prices generally ≤$20), 4) casual dining (offers sit-down/full table service; typically serves alcohol; entrée prices generally $15–$25), or 5) fine dining (entrée prices are generally >$25). These market segments are recognized industry standards, allowing for comparison across geographic regions. Restaurant cuisine type, coded by the market research firm on the basis of the primary type of food served by the restaurant, was categorized as American/Southern (bar and grill, diner, sports bar, brew pub), Asian (Chinese, Japanese, Korean, Thai, other Asian), Latino (Mexican, South American), coffee/bakery/dessert (bagel, coffee shop, ice cream, smoothie, donut), burger, pizza, sandwich/deli, European (Italian, Mediterranean, French, other European), or other (African, Caribbean, Indian, seafood, mixed ethnicity, steakhouse, barbecue).

We selected neighborhood characteristics on the basis of population groups that tend to be disproportionately affected by diet-related diseases. We collected the following census tract–level data from the 2010–2014 American Community Survey 5-Year Estimates (19–23): 1) percentage of non-Hispanic white residents; 2) percentage of the population aged 25 or older with more than a high school education; 3) the percentage of the population with income below the poverty level in the last 12 months; 4) median household income in the last 12 months, in 2014 inflation-adjusted dollars; and 5) total population.

### Data cleaning, geocoding, and aggregation

The original data set contained 24,884 restaurants, as of September 19, 2016. Staff members of the Los Angeles County Department of Public Health (DPH) conducted a 3-stage cleaning and geocoding process. First, they flagged possible duplicate records on the basis of similarities in restaurant name, street address, and/or telephone number. Second, they flagged possible unidentified chain locations on the basis of similarities in restaurant name and telephone number. All flags were investigated via internet search. Third, they geocoded restaurant addresses in ArcMap version 10.3.1 (ESRI) by using Los Angeles County’s Countywide Address Management System address locator (24). After cleaning, 24,292 restaurants remained in the final restaurant data set.

We defined Los Angeles County neighborhoods according to the *Los Angeles Times*’ Mapping L.A. project, which defines neighborhoods (a city, a community within a city, or an unincorporated area of the county) that are meaningful to residents and align with census-tract boundaries (25). We used the ArcMap Dissolve tool to aggregate census data to the neighborhood level. We constructed the following neighborhood-level measures of restaurant density: 1) total number of restaurants, 2) total number of restaurants per 1,000 residents (number of restaurants divided by the neighborhood population, multiplied by 1,000), 3) percentage of restaurants that were large chains (number of restaurants categorized as large chains [≥20 locations, based on the number of locations nationally or in Los Angeles County] divided by the number of total restaurants), 4) percentage of restaurants in each industry market segment (eg, quick service, fast casual), and 5) percentage of restaurants serving each type of cuisine (eg, American/Southern, Asian).

We excluded 8 neighborhoods that had fewer than 1,000 residents because the neighborhoods entirely or primarily were non-neighborhood–type complexes (eg, theme park, recreation area, health care campus). We excluded as outliers 3 neighborhoods that had more than 15 restaurants per 1,000 residents because they were large business or entertainment districts where daytime populations greatly exceed residential populations. Thus, we included 247 neighborhoods in the final neighborhood data set. In a sensitivity analysis of the relationship between restaurant chain density and neighborhood sociodemographic characteristics, we excluded 32 neighborhoods that had fewer than 10 restaurants. 

### Data analysis

We conducted descriptive and bivariate analyses at the restaurant level to examine the number and characteristics of restaurants countywide. We conducted descriptive and linear regression analyses at the neighborhood level to examine the association between neighborhood sociodemographic characteristics and 1) the number of restaurants per 1,000 residents and 2) the percentage of restaurants categorized as large chains. We conducted all analyses by using Stata version 14.1 (StataCorp LP). All materials were reviewed and approved by the Los Angeles County Department of Public Health Institutional Review Board.

## Results

The final sample consisted of 24,292 restaurants in Los Angeles County. On the basis of the number of locations nationally, we classified 26.5% of restaurants as large chain, 11.3% as small chain, and 62.2% as independent (Table 1). The 6,430 restaurant locations categorized as large chains represented 278 restaurant brands. On the basis of the number of locations in Los Angeles County, we classified 21.2% restaurants as large chain. The 5,145 locations categorized as large chains represented only 59 brands. These 59 brands represented 80% of all large chain restaurants in the county.

**Table 1:** Characteristics of Restaurants (N = 24,292) in Los Angeles County, California, 2016^a^

Characteristic	Number (%)
**Chain status **	
Based on number of locations nationally	
Independent (single location)	15,114 (62.2)
Small chain (2–19 locations)	2,748 (11.3)
Large chain (≥20 locations)	6,430 (26.5)
Based on number of locations in Los Angeles County	
Independent (single location)	15,449 (63.6)
Small chain (2–19 locations)	3,698 (15.2)
Large chain (≥20 locations)	5,145 (21.2)
**Industry market segment**	
Quick service	9,571 (39.4)
Fast casual	2,548 (10.5)
Midscale dining	4,626 (19.0)
Casual dining	6,483 (26.7)
Fine dining	609 (2.5)
Missing data	455 (1.9)
**Type of cuisine**	
American/Southern	4,476 (18.4)
Asian	4,438 (18.3)
Latino	3,765 (15.5)
Coffee/bakery/dessert	3,208 (13.2)
Burger	1,943 (8.0)
Pizza	1,692 (7.0)
Sandwich/deli	1,566 (6.5)
European	1,520 (6.3)
Other	1,493 (6.1)
Missing data	191 (0.8)

a Information on restaurant characteristics was provided by a market research firm that tracks restaurant industry trends nationally.

Large chain restaurants were more likely than other types of restaurants to be quick service or fast casual. On the basis of the number of locations nationally, 66.2% of large chains were quick service and 21.5% were fast casual, compared with 29.9% and 5.7% of independent restaurants that were quick service or fast casual, respectively. Large chains were most likely to serve coffee/bakery/dessert (22.4%), burger (19.7%), and sandwich (14.2%) cuisines. Independent restaurants were most likely to serve Asian (23.4%), American/Southern (21.5%), or Latino (17.0%) cuisines.

Although most large chains were classified as quick service or fast casual restaurants, not all quick service and fast casual restaurants were classified as large chains. Among quick service restaurants (n = 9,571), less than half (44.5%) were classified as large chains on the basis of the number of locations nationally.

### Neighborhood sociodemographic characteristics and restaurant density

The average number of restaurants, by neighborhood, was 94.4 (standard deviation [SD], 117.5) (Table 2). The number of restaurants ranged from 0 (4 neighborhoods) to more than 500 restaurants (4 neighborhoods); the median was 58 (interquartile range, 101). The average number of restaurants was 2.3 (SD, 1.8) per 1,000 residents but ranged from 0 to 11.6. The median was 1.9 (interquartile range, 1.6).

**Table 2:** Characteristics of Neighborhoods (N = 247) in Los Angeles County, California, 2016^a^

Characteristic	Mean (Standard Deviation)
**Sociodemographic characteristics^b^**	
Total population	40,210 (43,989)
Race/ethnicity	
Percentage of Non-Hispanic white residents	31.5 (26.4)
Percentage of Hispanic or Latino residents	43.4 (27.6)
Percentage of black or African American residents	8.7 (14.3)
Percentage of Asian residents	13.5 (14.1)
Percentage of residents aged ≥25 with >high school education	58.0 (21.6)
Percentage of residents below the poverty level in the last 12 months	16.7 (10.0)
Median household income in the last 12 months, $	67,895.7 (31,671.8)
**Restaurant characteristics**	
Number of restaurants	94.4 (117.5)^c^
Number of restaurants per 1,000 residents	2.3 (1.8)
Proportion of restaurants that are large chains based on the number of locations nationally	26.5 (15.0)^d^
Proportion of restaurants that are large chains based on the number of locations in Los Angeles County	21.7 (12.4)^e^

a Neighborhoods and their boundaries were defined according to the *Los Angeles Times*’ Mapping L.A. project. All analyses excluded 8 neighborhoods with <1,000 residents and 3 neighborhoods with >15 restaurants per 1,000 residents.

b Based on census tract level data drawn from the 2010–2014 American Community Survey 5-Year Estimates, aggregated to the neighborhood level (19–23).

c Thirty-two of 247 neighborhoods (13.0%) had <10 restaurants and were excluded in sensitivity analyses examining the relationship between restaurant chain density and neighborhood sociodemographic characteristics.

d When we examined neighborhoods with ≥10 restaurants (n = 215), average was 27.2% and standard deviation was 12.5%.

e When we examined neighborhoods with ≥10 restaurants (n = 215), average was 22.9% and standard deviation was 10.5%.

Neighborhood education level, racial/ethnic composition, and poverty were significantly associated with the number of restaurants in the neighborhood. On average, for every 1-point increase in the percentage of residents with more than a high school education, the number of restaurants per 1,000 residents would be expected to increase by 2.5 (95% confidence interval [CI], 1.5–3.5). On average, for every 1-point increase in the percentage of non-Hispanic white residents in the neighborhood, the number of restaurants per 1,000 residents would be expected to increase by 1.9 (95% CI, 1.1–2.7). On average, for every 1-point increase in the percentage of residents below the poverty level, the number of restaurants per 1,000 residents would be expected to decrease by 3.3 (95% CI, −5.5 to −1.1). Median household income was not significantly associated with restaurant density.

### Neighborhood sociodemographic characteristics and restaurant characteristics

**Chain status.** On the basis of the number of locations nationally, the average density of large chain restaurants by neighborhood was 26.5% (SD, 15.0%), although the density of large chain restaurants ranged from 0% to 100% across neighborhoods. On the basis of the number of locations in Los Angeles County, the average density of large chain restaurants was 21.7% (SD, 12.4%); density ranged from 0% to 66.7%.

When we examined the number of locations of restaurants nationally, we found no significant associations between neighborhood sociodemographic characteristics and chain density. Neighborhoods with a greater density of large chains tended to have a lower percentage of non-Hispanic white residents and a lower percentage of residents with more than a high school education. When we examined the number of restaurant locations in Los Angeles County, we found significant associations between a greater density of large chain restaurants and a lower percentage of non-Hispanic white residents and a lower percentage of residents with more than a high school education (Table 3). Results did not substantively change in magnitude or significance when we considered only neighborhoods with 10 or more restaurants.

**Table 3:** Relationship Between Neighborhood Sociodemographic Characteristics and Density of Large Chain Restaurants, Los Angeles County, California, 2016^a^

Quartile	Mean (Standard Deviation)			
	Percentage of Non-Hispanic White Residents	Percentage of Residents with >High School Education	Percentage of Residents Below the Poverty Level	Median Household Income, $
**Percentage of restaurants that are large chain** b ^,^ c **(based on the number of locations nationally)**				
Quartile 1 (0%–18%)	36.0 (28.4)	60.9 (21.8)	17.0 (10.7)	68,360.7 (32,674.4)
Quartile 2 (19%–26%)	35.2 (28.8)	58.4 (24.0)	18.4 (9.9)	65,370.3 (32,879.2)
Quartile 3 (27%–35%)	26.8 (23.4)	56.1 (20.4)	15.8 (8.0)	65,832.0 (24,492.2)
Quartile 4 (>35%)	28.4 (24.2)	56.9 (20.4)	15.6 (11.0)	71,683.3 (35,499.6)
**Percentage of restaurants that are large chain** b ^,^ c **(based on the number of locations in Los Angeles County)**				
Quartile 1 (0%–14%)	40.5 (28.8)	64.4 (21.7)	15.4 (10.2)	74,177.7 (36,175.4)
Quartile 2 (15%–22%)	34.2 (28.0)	59.2 (24.1)	18.1 (11.1)	66,282.7 (30,176.5)
Quartile 3 (23%–29%)	27.3 (22.3)^d^	56.0 (18.4)^e^	16.0 (7.0)	64,073.5 (21,896.4)
Quartile 4 (>29%)	24.8 (23.9)^f^	53.0 (20.8)^g^	17.3 (11.0)	67,197.8 (35,946.7)

a Neighborhoods (N = 247) and their boundaries were defined according to the *Los Angeles Times*’ Mapping L.A. project. All analyses excluded 8 neighborhoods with <1,000 residents and 3 neighborhoods with >15 restaurants per 1,000 residents.

b Results did not substantively or significantly change when analyses were conducted on neighborhoods with ≥10 restaurants (n = 215).

c Large chain restaurants were defined as restaurants with ≥20 locations.

d *P* = .005 for difference between quartile 3 and quartile 1, based on simple linear regression.

e *P* = .03 for difference between quartile 3 and quartile 1, based on simple linear regression.

f *P* = .001 for difference between quartile 3 and quartile 1, based on simple linear regression.

g *P* = .003 for difference between quartile 3 and quartile 1, based on simple linear regression.

**Restaurants by industry market segment and cuisine.** We found high correlations between the proportion of residents with more than a high school education and the proportion of 1) quick-service restaurants (*r* = −0.42) and 2) fine-dining restaurants (*r* = 0.46). In neighborhoods with the lowest quartile of residents with more than a high school education, roughly half of the restaurants were quick service (mean = 0.49; SD, 0.13), and in neighborhoods with the highest quartile of residents with more than a high school education, approximately one-third (mean = 0.32; SD = 0.14) of restaurants were quick service. We found correlations and proportions of similar magnitude between these 2 restaurant segments and the percentage of non-Hispanic white residents.

For cuisine type, we found high correlations between the proportion of non-Hispanic white residents and the proportion of restaurants that served European cuisine (*r* = 0.69) and Latino cuisine (*r* = −0.51). The proportion of residents with more than a high school education was strongly correlated with the proportion of restaurants that served Latino cuisine (*r* = −0.69), European cuisine (*r* = 0.58), coffee/bakery/dessert cuisine (*r* = 0.44), and burger cuisine (*r* = −0.43).

## Discussion

Our study suggests that a limited proportion of restaurants in Los Angeles County are part of a large chain. A policy that targets large chains (≥20 locations nationally) would affect only about one-quarter of all restaurants in Los Angeles County. Estimates of chain density in our study are lower than national estimates, which suggest that 40% of all restaurants are part of a large chain (3). Our study highlights the potentially limited reach of policy strategies (such as menu labeling) that would target chain restaurants in Los Angeles County and point to the importance of reaching out to and collaborating with small chain restaurants and independent restaurants as part of a comprehensive local strategy to improve the healthfulness of restaurants. The importance of targeting such restaurants is underscored by recent work demonstrating that non-chain restaurants offer high-calorie food, on par with their chain counterparts (26). Independent and small chain restaurants may face challenges to participating in restaurant-based strategies, especially when recipe analysis or sales tracking is required. To address these barriers, lessons may be drawn from work with small food store owners, who often struggle to stock healthy items because of limitations related to infrastructure, staff expertise, and access to appropriate suppliers (27).

In general, we found more restaurants in neighborhoods that had a greater percentage of non-Hispanic white residents and residents with more than a high school education; these populations tend to be less affected by diet-related diseases. Where restaurants choose to locate is driven by various market forces, including the proportion of targeted households, traffic generators, and sales generators (28). Given that spending on food purchased away from home increases with income (29), we were not surprised that the density of restaurants was greater in Los Angeles County’s higher-income neighborhoods, where income level might allow for a greater amount of discretionary spending than in lower-income neighborhoods. Few studies have examined restaurant density (as a whole) in relationship to neighborhood demographic characteristics. One national study showed that higher-income neighborhoods and neighborhoods with predominantly black or African American and racially mixed residents had lower levels of access to both fast-food and full-service restaurants (16). Previous work in Los Angeles County found that lower-middle and upper-middle socioeconomic census tracts had the highest total number of restaurants, compared with very low- and very high-income tracts (14). Previous literature does not clarify whether a greater density of restaurants is protective or detrimental. Greater restaurant density could mean more consumer choice to seek out healthy options. Alternatively, given that consuming food at restaurants is associated with greater intake of energy, fat, and sodium, relative to consuming foods prepared at home (30), greater restaurant density could lead to less healthy behaviors and outcomes.

Our study provides limited support to the idea that large chains are more heavily concentrated in certain neighborhoods (neighborhoods with a lower percentage of non-Hispanic white residents and a lower percentage of residents with more than a high school education). We did not observe significant relationships between chain density and neighborhood sociodemographic characteristics when chains were defined according to the number of locations nationally; rather, we observed relationships between chain density and race/ethnicity and education level only when we examined restaurants with 20 or more locations in Los Angeles County. Previous research on the relationship between density of chain restaurants and neighborhood sociodemographic characteristics is limited, and these studies tended to conflate chain status with fast-food service style and cuisine. In our study, although many chain restaurants were classified as quick service (fast food), not all quick service restaurants were part of a chain.

Although the completeness and large sample sizes of the data sources are strengths, our study has several limitations. First, our analysis does not provide any information on the reasons or causal mechanisms underlying the observed associations between community characteristics and restaurant density and characteristics. Restaurant location is driven by various market forces; restaurants often choose to cluster in commercial areas. Second, we used sociodemographic indicators as rough markers of disproportionate disease burden. Mapping the restaurant landscape in relationship to the prevalence of diet-related disease is an important area for future work. Third, although people are likely to visit restaurants outside their neighborhood, our study treated neighborhood boundaries as rigid boundaries and did not account for travel distance to restaurants or the profile of restaurants near a person’s school or work. For our analysis to have useful and relevant neighborhood boundaries, we used neighborhood definitions that were meaningful to residents, while respecting census boundaries to accurately integrate demographic data. However, calculating restaurant density according to census units did not account for edge effects, particularly along major arterials. Finally, the market research data did not provide any information on the relative healthfulness, size, or sales of restaurants. We used market research data rather than administrative data because they provided a more accurate picture of the number and types of restaurants, particularly information on chain status (the major study question). Future studies would benefit from data sets that include information on the relative healthfulness of food options.

Our study provides insight on the potential importance of including small chain and independent restaurants in efforts to advance healthier food access in communities. The extent to which restaurant-based strategies are an effective means to target diet-related disparities remains unclear. Additional work is needed to better understand the extent to which restaurant-based initiatives can effectively reach people most in need. It is important to consider where restaurants — especially those affected or targeted by program or policy work (such as chains) — are located in relation to priority populations. Our study answers a question that is infrequently examined yet of critical importance to advance local public health practice. Applied researchers and evaluators in other jurisdictions can build on the methods used in our study to gain a deeper understanding of their local landscape.
